# Prothrombin G20210A (rs1799963) polymorphism increases myocardial infarction risk in an age-related manner: A systematic review and meta-analysis

**DOI:** 10.1038/s41598-017-13623-6

**Published:** 2017-10-19

**Authors:** Changlong Li, Hui Ren, Hong Chen, Junxian Song, Sufang Li, Chongyou Lee, Jun Liu, Yuxia Cui

**Affiliations:** 1 Department of Cardiology, Peking University People’s Hospital, Beijing, China; 2 Beijing Key Laboratory of Early Prediction and Intervention of Acute Myocardial Infarction, Peking University People’s Hospital, Beijing, China; 3 Center for Cardiovascular Translational Research, Peking University People’s Hospital, Beijing, China

## Abstract

G20210A polymorphism (rs1799963) within the prothrombin gene is associated with a higher circulation level of prothrombin, thus increasing the likelihood of developing myocardial infarction (MI). Opinions differ regarding the correlation between prothrombin G20210A genotype and MI risk, which prompted us to conduct a meta-analysis to determine this association. PubMed, EMBASE, Web of Science and CNKI were searched for pertinent reports. A total of 34 studies involving 14 611 MI cases and 84 358 controls were analyzed in this quantitative analysis. We found a statistically significant association between prothrombin G20210A polymorphism and MI in the allele model (A vs. G, OR = 1.43, 95%CI: 1.18–1.72), heterozygote model (GA vs. GG, OR = 1.41, 95%CI: 1.16–1.72) and dominant model (GA + AA vs. GG, OR = 1.41, 95%CI: 1.15–1.72). The association remains significant in Caucasians but not in non-Caucasians. Moreover, prothrombin G20210A polymorphism increases MI risk in an age-related manner. A further significant association was found in a subpopulation younger than 55 years (allele model, OR = 1.76, 95%CI: 1.32–2.35; heterozygote model, OR = 1.70, 95%CI: 1.24–2.33; dominant model, OR = 1.70, 95%CI: 1.24–2.34). Sensitivity analysis and publication bias analysis revealed stable and statistically robust results. Our meta-analysis demonstrated that prothrombin G20210A polymorphism may represent a risk factor for MI.

## Introduction

Myocardial infarction (MI) is a highly lethal disease in developed countries, usually caused by pathological coronary artery occlusion. However, unstable atherosclerotic plaque is not destined to cause MI. Following plaque rupture, coronary thrombosis involving platelet adhesion, coagulation cascade activation and subsequent thrombus formation plays a fundamental role in the clinical progression to MI. Over-activated procoagulation, acquired from complex patterns of inheritance or environmental risk factors, has more propensity to induce localized coronary occlusion. One of the most concerning etiological factors is single-nucleotide polymorphisms (SNPs) in coagulation and fibrinolytic systems.

Prothrombin, also called factor II, is the precursor of the thrombin, a key enzyme acting as a procoagulant, through platelet activation and the generation of fibrin and factors Va, VIIIa, and XIII^1^. Numerous studies have demonstrated that a G20210A variant (rs1799963) of the prothrombin gene (i.e. a G to A substitution at position 20 210 in the 3′-untranslated region) is associated with elevated circulation levels of prothrombin^2–4^ and risk of venous thromboembolism^2,5,6^. Nevertheless, the detrimental role of prothrombin G20210A is controversial in MI^7,8^. The reason for this confounding phenomenon may be diversity of the ages of patients recruited in the previous studies. Inherited defects in coagulation may contribute more to MI in the young than in the elderly^9^. In comparison to MI in the elderly, premature myocardial infarction might be more predisposed to a SNP such as prothrombin G20210A because of relatively less environmental exposure. To draw a conclusion about the clinical impact of the prothrombin G20210A with regard to MI risk within a given age stratum, we gathered data from a number of published studies and performed a meta-analysis.

## Results

### Characteristics of identified studies

**Figure 1:**
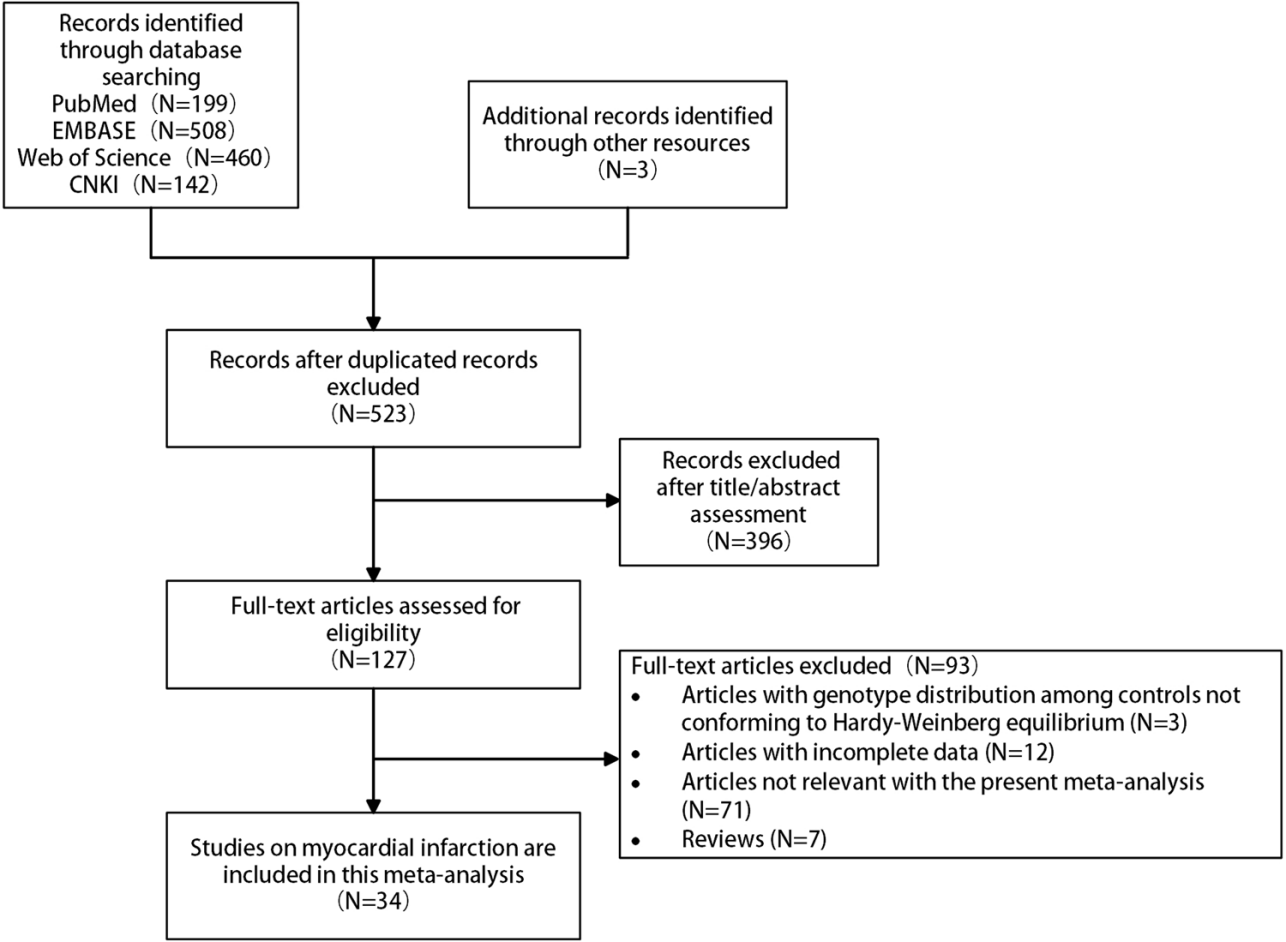
The flow chart of the included studies for the meta-analysis of prothrombin G20210A polymorphism and myocardial infarction risk.

As depicted in Fig. 1, 199 studies were searched in PubMed, 508 studies in EMBASE, 460 studies in Web of Science, and 142 studies in CNKI; 3 additional articles were selected from references in the reviews to make our search comprehensive. After excluding duplicated records, 523 published records were identified. Three hundred and ninety-six records were excluded after title/abstract assessment. Full text articles of the remaining 127 records were assessed for eligibility. After trying our best to communicate with the first and corresponding authors to obtain the necessary data, 34 studies were ultimately identified for our meta-analysis^4–6,8,10–39^.

There was one prospective study^6^ and 33 cross-sectional case–control studies. Twenty-two studies included Caucasian patients^4,6,11,13,14,16,18,20,21,23–27,29,33–39^, 9 included non-Caucasians^8,10,12,15,17,19,28,30,32^, and 3 did not depict ethnicity of subjects^5,22,31^. As the study population overlapped in two reports^6,39^, Maren’s data^39^ were evaluated only in the >55-year-old subgroup analysis and not in the overall analysis.

A total of 14 611 cases and 84 358 controls were included in the meta-analysis. Main characteristics, including first author, publication year, study country, ethnicity, age category of MI patients, source of controls, genotyping method, genotype frequencies, and minor allele frequencies are presented in Supplementary Table S1. Allele number, but not genotype frequency, was available in one study^27^, which consequently was included in the allele model analysis only. Because there was only one eligible study^19^ of homozygote and recessive models in non-Caucasian subgroup analysis, homozygote and recessive analyses were not performed in the non-Caucasian subgroup.

### Prothrombin G20210A and myocardial infarction

**Figure 2:**
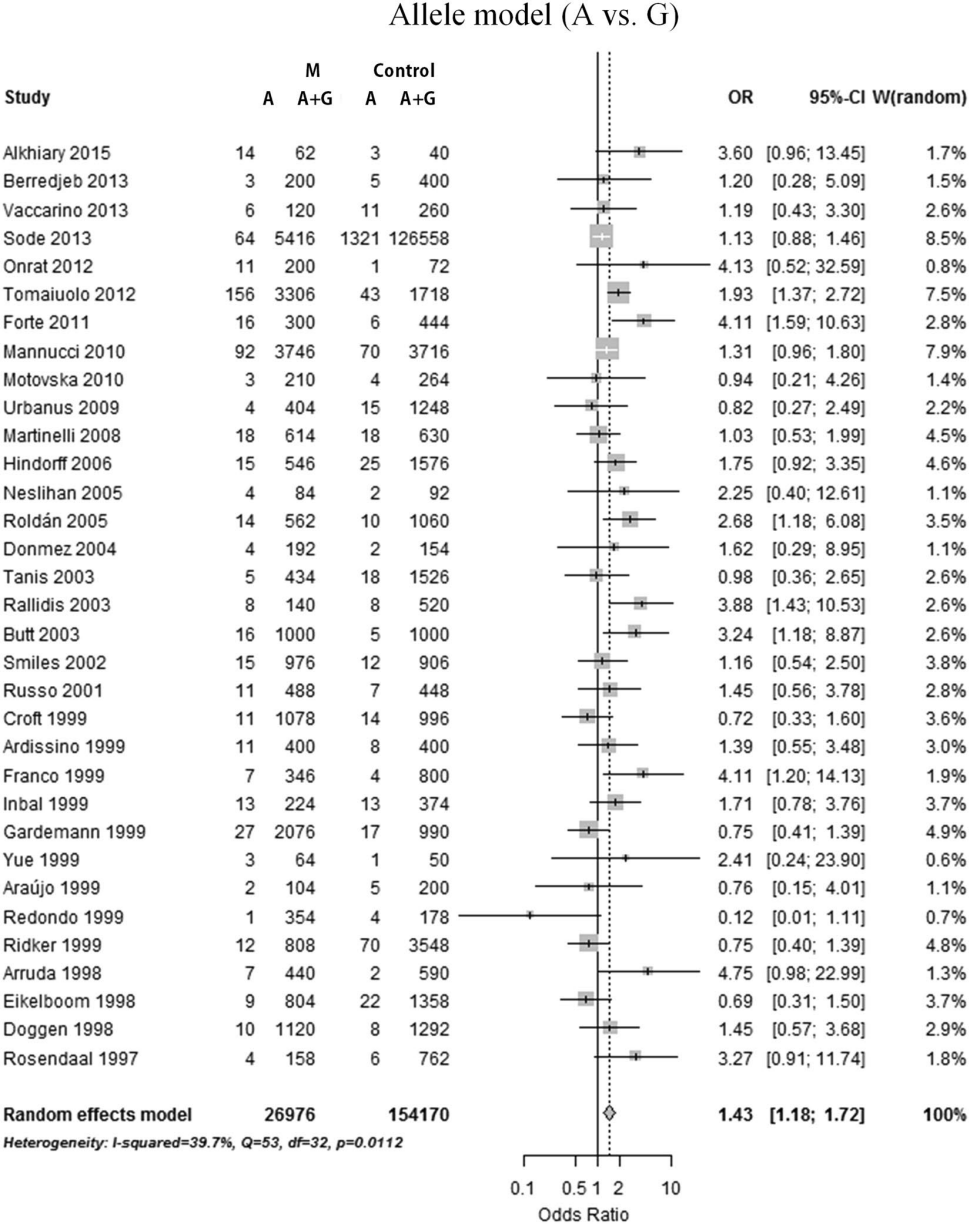
Forest plot for overall analysis of association of prothrombin G20210A polymorphism and myocardial infarction risk in an allele model (A allele vs. G allele). CI: confidence interval, OR: odds ratio, MI: myocardial infarction.

**Figure 3:**
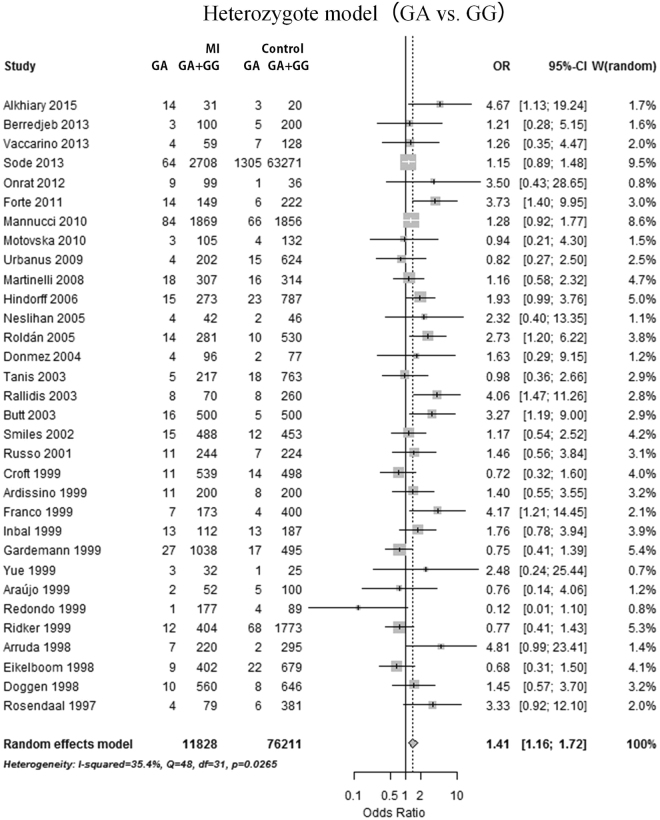
Forest plot for overall analysis of association of prothrombin G20210A polymorphism and myocardial infarction risk in a heterozygote model (GA vs. GG). CI: confidence interval, OR: odds ratio, MI: myocardial infarction.

**Figure 4:**
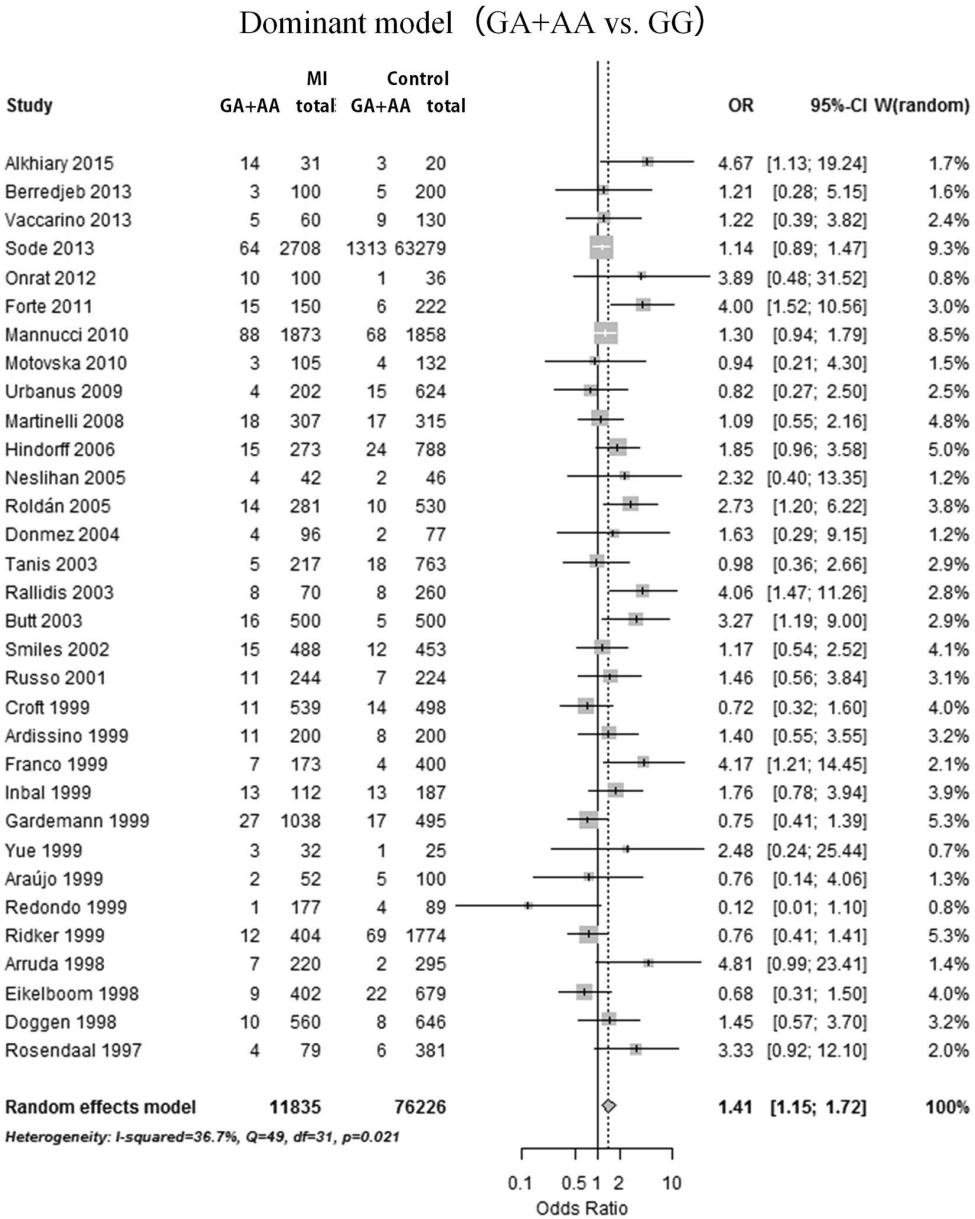
Forest plot for overall analysis of association of prothrombin G20210A polymorphism and myocardial infarction risk in a dominant model (GA + AA vs. GG). CI: confidence interval, OR: odds ratio, MI: myocardial infarction.

Overall, a meta-analysis of 13 488 cases and 77 085 controls in 33 studies was performed. The mean age was 48.41 years in cases and 47.16 years in controls. The association between prothrombin G20210A variant adenine (A) allele distribution and MI risk was calculated in comparison with wild-type guanine (G) allele in the allele model (A vs. G). Similarly, the association between specific genotype frequency and MI risk was assessed in the dominant model (AG + AA vs. GG), recessive model (AA vs. AG + GG), homozygote model (AA vs. GG), and heterozygote model (AG vs. GG), respectively. A statistically significant association was found in the allele model (mutation [A] allele versus wild-type [G] allele, random effects model [REM], odds ratio [OR] = 1.43, 95% confidence interval [95%CI]: 1.18–1.72, p = 0.0002; Fig. 2), heterozygote model (GA vs. GG, REM OR = 1.41, 95%CI: 1.16–1.72, p = 0.0007; Fig. 3) and dominant model (GA + AA vs. GG, REM OR = 1.41, 95%CI: 1.15–1.72, p = 0.0007; Fig. 4). However, no significant association was found in the homozygote (AA vs. GG, fixed effects model [FEM] OR = 1.42, 95%CI: 0.58–3.48, p = 0.45; Supplementary Figure S1) and recessive (AA vs. GA + GG, FEM OR = 1.39, 95%CI: 0.56–3.42, p = 0.48; Supplementary Figure S2) comparison. The results are summarized in Table 1 and Supplementary Table S2.

**Table 1:** Summary ORs for the Association of prothrombin G20210A SNP with Myocardial Infarction.

Variables		Overall	Ethnicity		Subgroups	
			Caucasian	Non-Caucasian	≤55 yo	>55 yo
Allele model	OR	REM 1.43	REM 1.40	FEM 1.51	REM 1.76	REM 1.43
	(95%CI)	(1.18–1.72)	(1.14–1.72)	(1.06–2.14)	(1.32–2.35)	(0.84–2.43)
	*p* value	0.0002	0.0012	0.022	0.0001	0.18
	I^2^ (%),	39.7	45.2	14.6	42.6	52.7
	P_het_	0.01	0.01	0.31	0.02	0.1
Homozygote model	OR	FEM 1.42	FEM 1.48	ND	FEM 1.77	FEM 3.45
	(95%CI)	(0.58–3.48)	(0.58–3.78)		(0.51–6.18)	(0.39–30.86)
	*p* value	0.45	0.41	ND	0.37	0.27
	I^2^ (%),	0	0	ND	0	0
	P_het_	0.98	0.95	ND	0.9	0.73
Heterozygote model	OR	REM 1.41	REM 1.37	FEM 1.51	REM 1.70	FEM 1.34
	(95%CI)	(1.16–1.72)	(1.11–1.70)	(1.05–2.15)	(1.24–2.33)	(0.98–1.84)
	*p* value	0.0007	0.0039	0.0244	0.0011	0.07
	I^2^ (%),	35.4	38.8	19.3,	36.4,	40.9
	P_het_	0.03	0.04	0.27	0.06	0.17
Dominant model	OR	REM 1.41	REM 1.37	FEM 1.52	REM 1.70	FEM 1.35
	(95%CI)	(1.15–1.72)	(1.10–1.69)	(1.06–2.16)	(1.24–2.34)	(0.99–1.85)
	*p* value	0.0007	0.0045	0.022	0.001	0.06
	I^2^ (%),	36.7,	40.1	20.8	36.8,	48.2,
	P_het_	0.02	0.03	0.26	0.05	0.12
Recessive model	OR	FEM 1.39	FEM 1.46	ND	FEM 1.73	FEM 3.31
	(95%CI)	(0.56–3.42)	(0.57–3.72)		(0.50–6.04)	(0.37–29.90)
	*p* value	0.48	0.43	ND	0.39	0.29
	I^2^ (%),	0	0	ND	0	0
	P_het_	0.98	0.95	ND	0.91	0.75

OR: odds ratios; CI: confidence interval; I^2^: I^2^ statistics; P_het_: Cochran’s Q statistics p-value for heterogeneity. REM: random effect model. FEM: fixed effect model. ND: no data.

In addition, prothrombin G20210A SNP was similarly associated with MI in three genetic models in Caucasians (allele model, REM OR = 1.40, 95%CI: 1.14–1.72, p = 0.0012; heterozygote model, REM OR = 1.37, 95%CI: 1.11–1.70, p = 0.0039; dominant model, REM OR = 1.37, 95%CI: 1.10–1.69, p = 0.0045) but not in non-Caucasians (allele model, FEM OR = 1.51, 95%CI: 1.06–2.14, p = 0.022; heterozygote model, FEM OR = 1.51, 95%CI: 1.05–2.15, p = 0.0244; dominant model, FEM OR = 1.52, 95%CI: 1.06–2.16, p = 0.022). Table 1 displays the results of overall and subgroup analyses.

### Prothrombin G20210A and risk of myocardial infarction before and after 55 years of age

Twenty reports^4,10–28^ (on 5 294 patients and 8 149 controls) provided data in subjects younger than 55 years. Four studies^14,32,38,39^ recruited 2 174 MI patients and 8 261 MI-free controls older than 55 years, respectively. The mean age was 41.75 years in cases and 42.03 in controls in the subgroup ≤55 years old, and 64.33 years in cases and 56.96 in controls in the subgroup >55 years old. Subgroup meta-analysis revealed that a significant association was found in three genetic models between prothrombin G20210A and MI in patients younger than 55 years (allele model, REM OR = 1.76, 95%CI: 1.32–2.35, p = 0.0001; heterozygote model, REM OR = 1.70, 95%CI: 1.24–2.33, p = 0.0011; dominant model, REM OR = 1.70, 95%CI: 1.24–2.34, p = 0.001) but not in individuals older than 55 (allele model, REM OR = 1.43, 95%CI: 0.84–2.43, p = 0.18; heterozygote model, FEM OR = 1.34, 95%CI: 0.98–1.84, p = 0.07; dominant model, FEM OR = 1.35, 95%CI: 0.99–1.85, p = 0.06) (Supplementary Figures S3–S8).

### Sensitivity analysis and publication bias

The independent studies were sequentially omitted to examine whether the recalculated ORs were significantly different in comparison with the originally combined effects. We found no obvious changes throughout the analysis, suggesting that our results are stable (Supplementary Figure S9).

**Figure 5:**
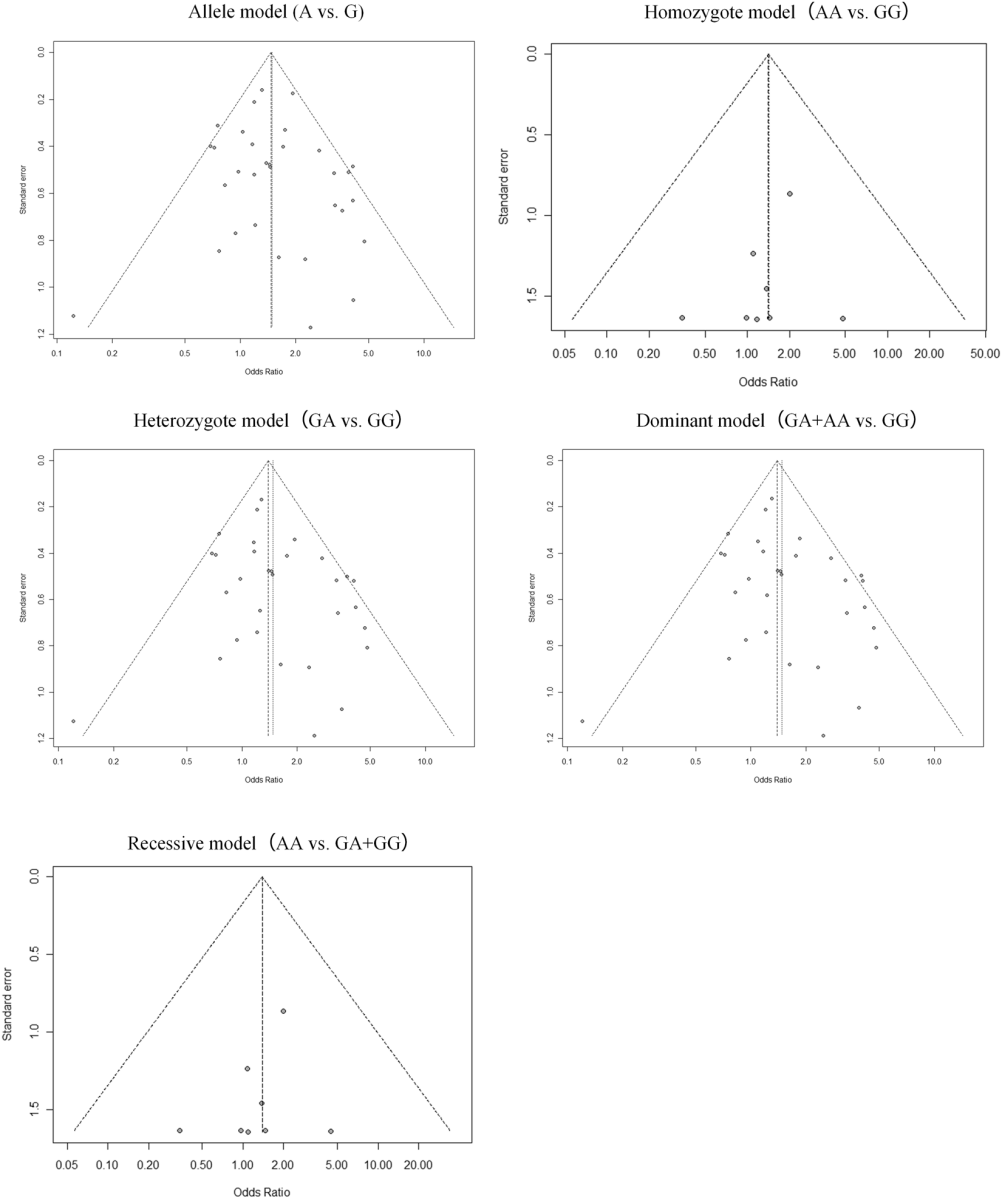
Assessment of publication bias on the relationships between G20210A polymorphism and susceptibility to MI with Begg’s funnel plots in five genetic models.

Publication bias of the studies was evaluated qualitatively by Begg’s funnel plot and Egger’s linear regression test. The p values were 0.28 (allele model), 0.62 (homozygote model), 0.13 (heterozygote model), 0.13 (dominant model), and 0.62 (recessive model) in Begg’s funnel plot (Fig. 5) and 0.27 (allele model), 0.43 (homozygote model), 0.09 (heterozygote model), 0.09 (dominant model), and 0.40 (recessive model) in Egger’s test (Supplementary Figure S10). Based on these results, no publication bias was found in our selected articles.

## Discussion

Myocardial infarction is pathologically caused by coronary thrombotic occlusion following rupture of atherosclerotic plaques. Prothrombin is the precursor of thrombin, which plays a pivotal role in the physiological process of coagulation. Located on chromosome 11, the G20210A variant (rs1799963) substitutes a single base of adenine (A) for guanine (G) at position 20 210 in the 3′-untranslated region of the prothrombin gene^2^. The prothrombin G20210A SNP has been correlated with increased levels of prothrombin in circulation and is clearly associated with an increased risk of venous thrombosis^2^. Substantive studies over the past decade have been making efforts to unmask the correlation between the G20210A SNP and MI. However, previous conflicting reports failed to draw a consistent conclusion, thereby making the association between the G20210A SNP and MI elusive^8,10,18,19,25–27,37–39^. The variance may result from different study designs and distinct ethnic backgrounds of subjects. Furthermore, several studies have reported that the observed association may be affected by other risk factors, such as age at onset. For instance, Butt *et al*.^13^ observed that the risk of MI was not elevated in prothrombin G20210A carriers beyond 50 years old, whereas carriers younger than 50 had a 5.6-fold higher risk of MI than age-matched non-carrier control subjects (p = 0.04). Thus, additional risk factors may influence the risk associated with this prothrombin G20210A variation. More importantly, inconsistency in the age of analyzed subjects may also cause discrepancy, as MI events in genetically vulnerable subjects occur much earlier than in normal counterparts^40^. Two previous systematic reviews^7,41^ analyzing the association between G20210A polymorphism and MI risk have drawn similar conclusions, namely that G20210A has no significant association with MI risk in the overall population at all ages, but does have a significant association with MI risk (OR = 1.77, 95%CI: 1.16–3.42) or risk tendency (OR = 1.86, 95%CI: 0.99–3.51) in persons younger than 55 years. To analyze whether MI is susceptible to prothrombin G20210A polymorphism, we performed this meta-analysis of 34 published studies to assess the association between prothrombin G20210A polymorphism and MI risk.

In the current systematic review we extracted allele and genotype frequency of the prothrombin G20210A polymorphism in MI patients and corresponding controls, and estimated the pooled ORs and 95%CI in five genetic models. Our integrated results showed that the G20210A polymorphism was significantly associated with MI risk in the allele model, heterozygote model, and dominant model. The tendency was similarly observed in Caucasians but not in non-Caucasians. The point estimates of the ORs in non-Caucasians, while not significant, are actually higher than those in Caucasians in three models. In subgroup analysis stratified by age, the MI risk in allele, heterozygote and dominant models was even higher in subjects ≤55 years old than in the overall population, but was not statistically significant in those older than 55.

In the present meta-analysis of 34 studies we drew the conclusion that prothrombin G20210A SNP was significantly associated with overall MI risk at all ages, contrasting with results from two previous independent meta-analysis reports^7,41^. Boekholdt *et al*.^41^ evaluated 4 studies and concluded that G20210A polymorphism was not significantly associated with MI risk (OR = 0.89, 95%CI: 0.59–1.35). Burzotta *et al*.^7^ assessed 13 studies and produced similarly insignificant results (OR = 1.19, 95%CI: 0.93–1.58). This inconsistency between prior and present outcomes may have at least two possible explanations. Firstly, the two previous meta-analyses enrolled 4 and 13 studies, respectively, whereas 34 studies are included herein. These additional studies with larger numbers of subjects may have enhanced the soundness of the conclusion. Secondly, there may be an age discrepancy between the present and previous meta-analyses. For instance, MI patients younger than 55 years accounted for 16.9% of the total (624/3 687) in Burzotta’s meta-analysis^7^, whereas in the present study this proportion was 39.2% (5 294/13 488). According to age-related subgroup analysis in previous reviews^7,41^ and the present study, prothrombin G20210A polymorphism markedly increases MI risk in younger people. The discrepancy in age may help to explain the disagreeing conclusions.

An important conclusion from the present research is that G20210A polymorphism correlated with MI in young patients but not in the elderly, consistent with the previous meta-analysis^7^. Premature myocardial infarction (PMI) commonly indicates a first onset of myocardial infarction before the age of 55 years in males and 65 years in females. Considering less exposure to long-term environmental risk factors, MI events in young individuals are more likely attributable to genetic susceptibility than those in elderly counterparts^40^. The dominant role of acquired risk factors in MI, such as hypercholesterolemia and smoking, attenuates the effects of genetic prothrombin G20210A in the elderly^9^. Several genetic mutations affecting coagulation proteins have been suggested as PMI risk factors^10,18,19^.

The association between prothrombin G20210A polymorphism and susceptibility to MI has been carefully investigated. Notwithstanding, in addition to the inevitable clinical heterogeneity in most systematic reviews, we must acknowledge some limitations of this meta-analysis. (1) We cannot exclude the potential for a survival bias. Most of the included studies were designed to recruit survivors of MI as a case group. It cannot be excluded that patients who had died during the acute phase of fatal MI more often carried the prothrombin G20210A allele mutation. A necropsy study^42^ of 33 cases of fatal MI and 165 controls showed a noticeable trend toward an association between the GA genotype and fatal MI (OR = 7.0, 95%CI: 0.6–82). (2) Statistically significant association between prothrombin G20210A and MI risk was not observed in the homozygote model (AA vs. GG OR = 1.28, 95%CI: 0.48–3.44) and the recessive model (AA vs. GG + GA OR = 1.26, 95%CI: 0.47–3.38), which seemed to conflict with the findings in the allele model (OR = 1.43, 95%CI: 1.18–1.72), heterozygote model (OR = 1.41, 95%CI: 1.16–1.72) and dominant model (OR = 1.41, 95%CI: 1.15–1.72). The reason for this conflict may be that AA genotype frequency in the overall cases and controls was extremely low (0.07% and 0.04%), which resulted in a population inadequate for researching the association with MI risk. Although two independent researchers searched the databases for published articles and recorded data from eligible studies, it was possible that some articles might not be included in the present study because: (a) the possibility of publication bias could not be eliminated even though no evidence of publication bias was detected through Begg’s funnel plot and Egger’s linear regression method; (b) the language was restricted to English and Chinese. The relatively small sample size and number of included studies for gene-susceptibility investigation might distort the stability of the results regarding homozygote and recessive models. The true detrimental role of homozygote genotype AA in MI may need to be proved in studies with a larger population. (3) Moreover, a potential age discrepancy existed in the overall population analyzed, as there was a greater proportion of young individuals. Subpopulations younger than 55 years were available in 20 studies (containing 5 294 cases and 8 149 controls), whereas those older than 55 were from only 4 studies (containing 2 174 cases and 8 261 controls). The mortality rate of MI in young patients is far less than that in elderly patients, demonstrating the possibility that more elderly patients are lost through fatal MI^43^.

In conclusion, this comprehensive meta-analysis of 34 studies shows evidence supporting a causal association between prothrombin G20210A SNP and vulnerability to myocardial infarction. The risk was higher in the population younger than 55 years, but was not observed in the subjects older than 55. Larger population studies are urgently needed to validate our current findings and address the pathogenic role, which remains poorly understood.

## Methods

### Study selection

The PRISMA statement was followed in this meta-analysis^44^. PubMed, EMBASE, Web of Science and China National Knowledge Infrastructure (CNKI) databases were searched from inception to 30 April, 2016 without language restriction. We used the combination of the search terms: (“atherosclerotic heart disease” OR “coronary artery disease” OR “CAD” OR “MI” OR “Myocardial Infarction” OR “AMI” OR “Acute Myocardial Infarction” OR “ACS” OR “Acute Coronary Syndrome”) AND (“polymorphism” OR “polymorphisms” OR “mutation” OR “gene polymorphism” OR “SNP” OR “Single Nucleotide Polymorphism” OR “genotypes” OR “genotype” OR “variants” OR “variant”) AND (”Prothrombin” OR “Factor II”). The searches were complemented by checking the references of the retrieved articles.

Eligibility criteria included the following: (1) case-control studies focused on association between prothrombin G20210A and myocardial infarction; (2) at least 30 patients were recruited in the case group; (3) when recruited patients overlapped with those in another study, the study with the largest number of individuals was selected; (4) if a study did not meet the inclusion criteria but a subgroup of the subjects qualified, only the subgroup was included; (5) studies that did not provide sufficient data following contact with authors by e-mail were excluded; (6) case reports and reviews were excluded. There was no notable deviation from Hardy–Weinberg equilibrium (HWE) in the control group.

### Data extraction and quality assessment

Two independent researchers (J.S. and S.L.) separately assessed the quality of the included studies using the Newcastle–Ottawa Scale (NOS) criteria^45^. Studies that were awarded 5 stars or more, considered as of medium to high quality, were included in the present study. The following information in studies was extracted by two independent researchers: first author, year of publication, ethnicity, country, age of MI patients, source of controls, genotyping method, genotype frequencies, number of cases and controls analyzed, mean value of age.

### Statistical analysis

R programming language (version 2.13.1) was used to analyze the selected data. The pooled ORs were calculated for the allele model (mutation [A] allele versus wild-type [G] allele), dominant model (AG + AA vs. GG), recessive model (AA vs. AG + GG), homozygote comparison (AA vs. GG), and heterozygote comparison (AG vs. GG), respectively. After adjusting for multiple testing with Bonferroni’s method, a p value of any comparison less than 0.01 (=0.05/5) was considered statistically significant. When calculating the OR in one genetic model, we excluded studies with no events in either MI or control groups. When only one group contained no events, we added 0.5 to each cell of the 2 × 2 table for the trial before analysis. HWE for each study was determined by Chi-square test.

Tests for study heterogeneity were performed using Higgins’ I^2^ statistics and Cochran’s Q statistics for each meta-analysis. I^2^ more than 50% or a p value less than 0.1 was considered heterogeneous among included studies. In such a case, the REM was used to summarize the values for each study, otherwise the FEM was applied. Begg’s and Egger’s tests were performed to assess publication bias, whereby a p value of less than 0.05 indicates an existence of publication bias among included studies.

## Electronic supplementary material

Supplementary Information
