# Supplementary material for: Prothrombin G20210A (rs1799963) polymorphism increases myocardial infarction risk in an age-related manner: A systematic review and meta-analysis
Source: Sci Rep. 2017 Oct 19;7:13550. doi: 10.1038/s41598-017-13623-6 (PMC5648836; doi:10.1038/s41598-017-13623-6)
Supplement: Supplementary file 1 — Supplementary Information [file 41598_2017_13623_MOESM1_ESM.pdf]

Prothrombin G20210A (rs1799963) polymorphism increases myocardial infarction risk in an age-related manner: A systematic review and meta-analysis

Changlong Li[1.2.3#], Hui Ren[1.2.3#], Hong Chen[1.2.3*], Junxian Song[1.2.3], Sufang Li[1.2.3], Chongyou Lee[1.2.3], Jun Liu[1.2.3], Yuxia Cui[1.2.3]

[1] Department of Cardiology, Peking University People's Hospital, Beijing, China.

[2] Beijing Key Laboratory of Early Prediction and Intervention of Acute Myocardial Infarction, Peking University People's Hospital, Beijing, China.

[3] Center for Cardiovascular Translational Research, Peking University People's Hospital, Beijing, China

# These authors contributed equally to the present work.

* Corresponding author, e-mail: chenhongbj@medmail.com.cn.

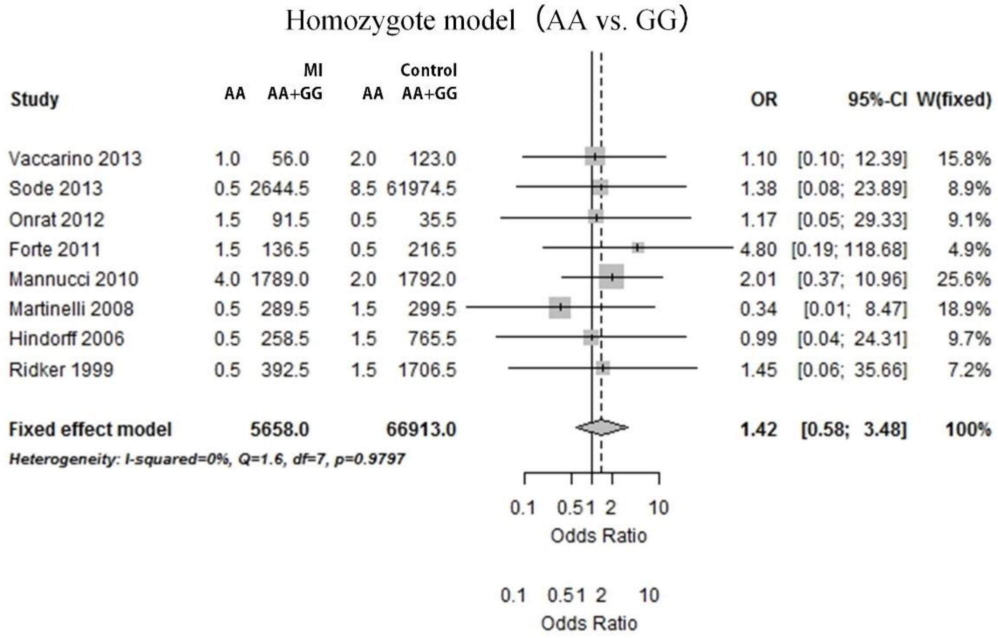

Supplementary Figure S1. Forest plot for overall analysis of association of prothrombin G20210A polymorphism and myocardial infarction risk in a homozygote model (AA vs. GG). CI: confidence interval, OR: odds ratio, MI: myocardial infarction.

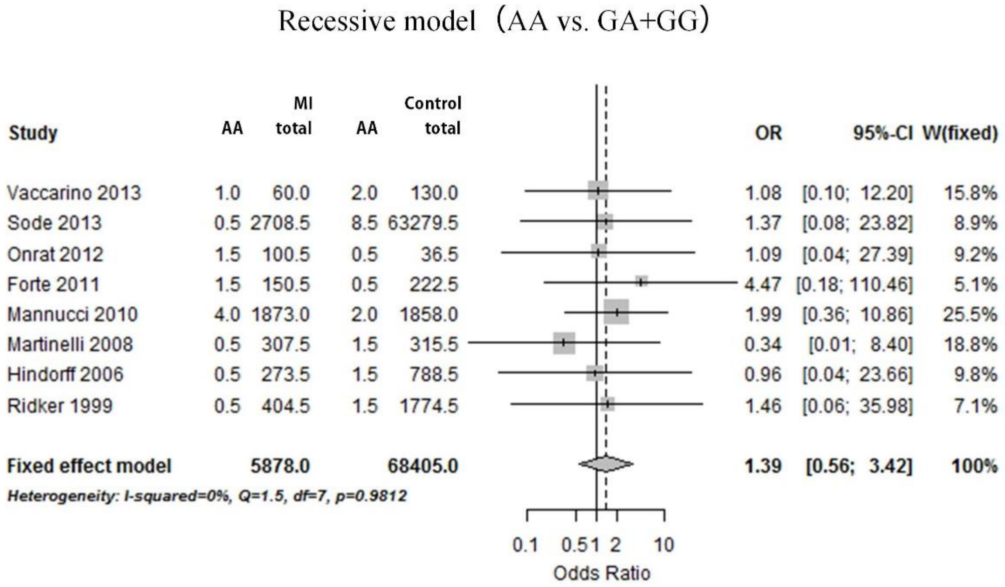

Supplementary Figure S2. Forest plot for overall analysis of association of prothrombin G20210A polymorphism and myocardial infarction risk in a recessive model (AA vs. GA+GG) . CI: confidence interval, OR: odds ratio, MI: myocardial infarction.

Allele model (A vs. G, <55)

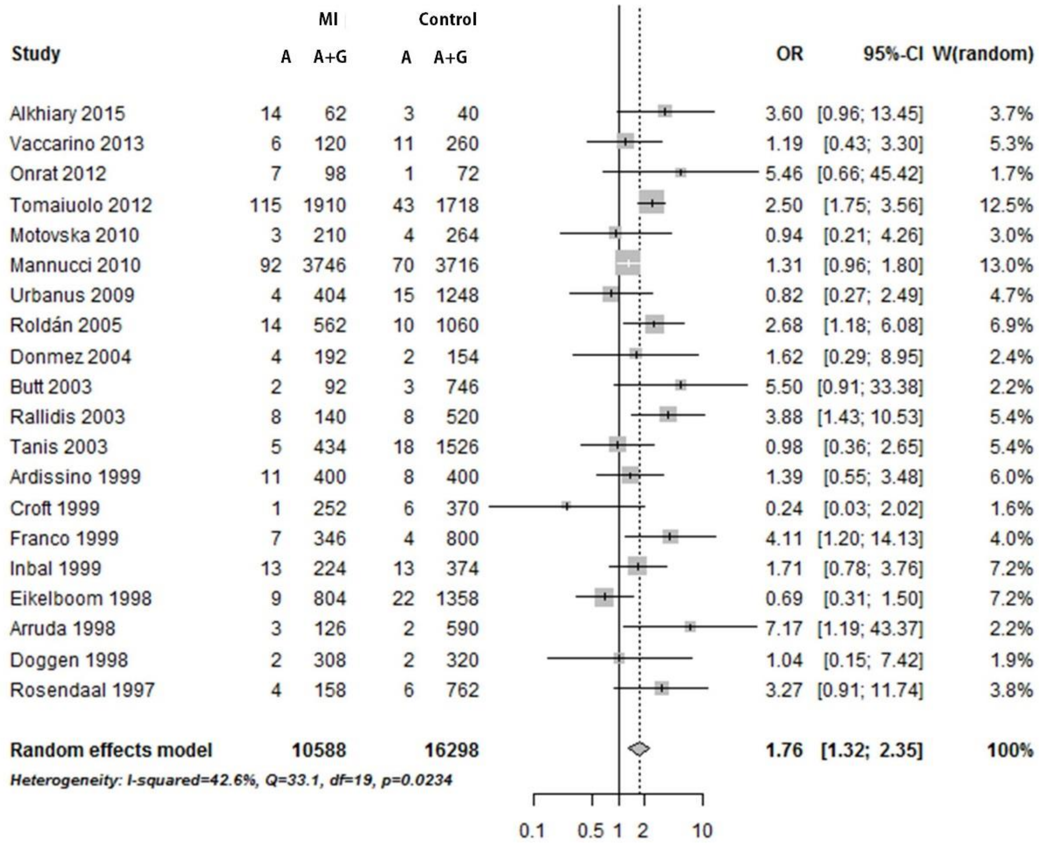

Supplementary Figure S3. Forest plot for subgroup younger than 55 years analysis of association of prothrombin G20210A polymorphism and myocardial infarction risk in an allele model(A allele vs. G allele). CI: confidence interval, OR: odds ratio, MI: myocardial infarction.

Homozygote model（AA vs. GG, <55）

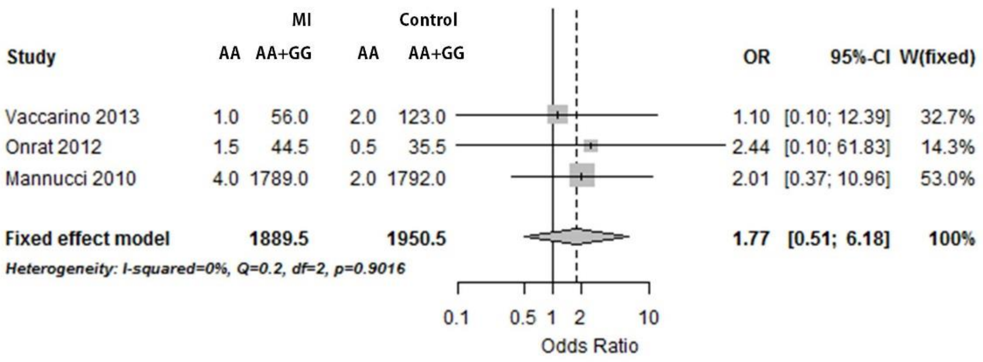

Supplementary Figure S4. Forest plot for subgroup younger than 55 years analysis of association of prothrombin G20210A polymorphism and myocardial infarction risk in a homozygote model (AA vs. GG). CI:confidence interval, OR:odds ratio, MI: myocardial infarction.

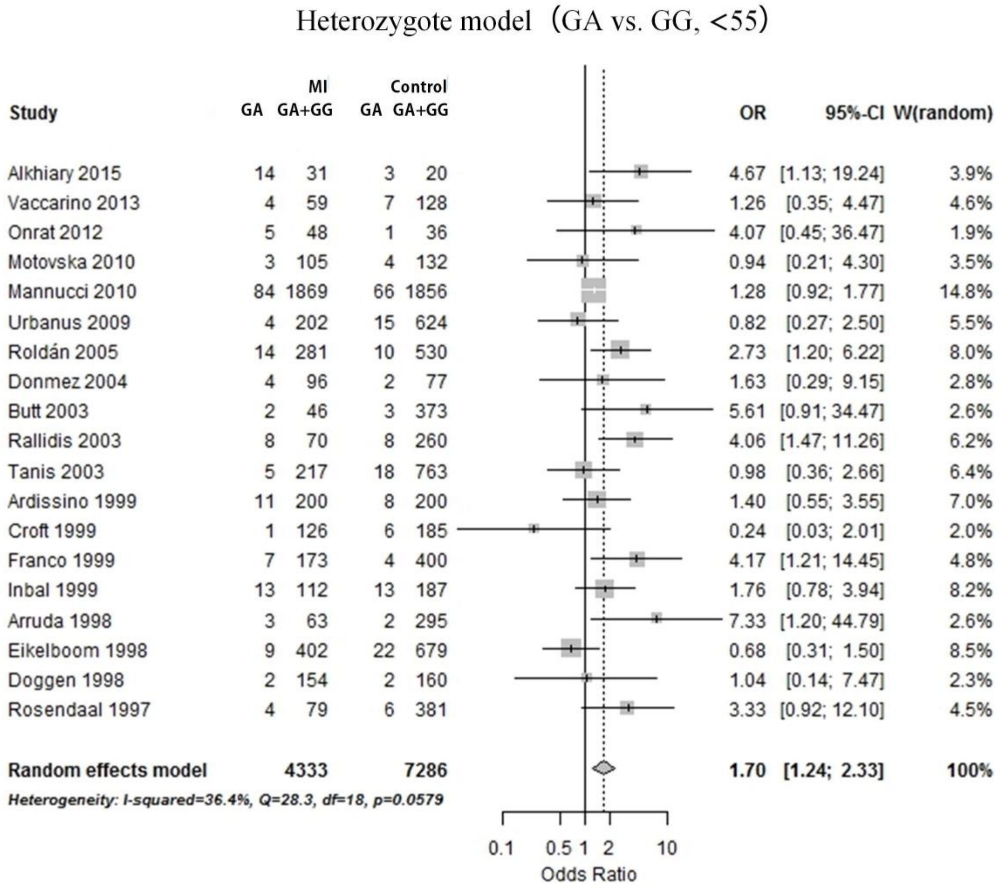

Supplementary Figure S5. Forest plot for subgroup younger than 55 years analysis of association of prothrombin G20210A polymorphism and myocardial infarction risk in a heterozygote model (GA vs. GG). CI:confidence interval, OR:odds ratio, MI: myocardial infarction.

Dominant model（GA+AA vs. GG, <55）

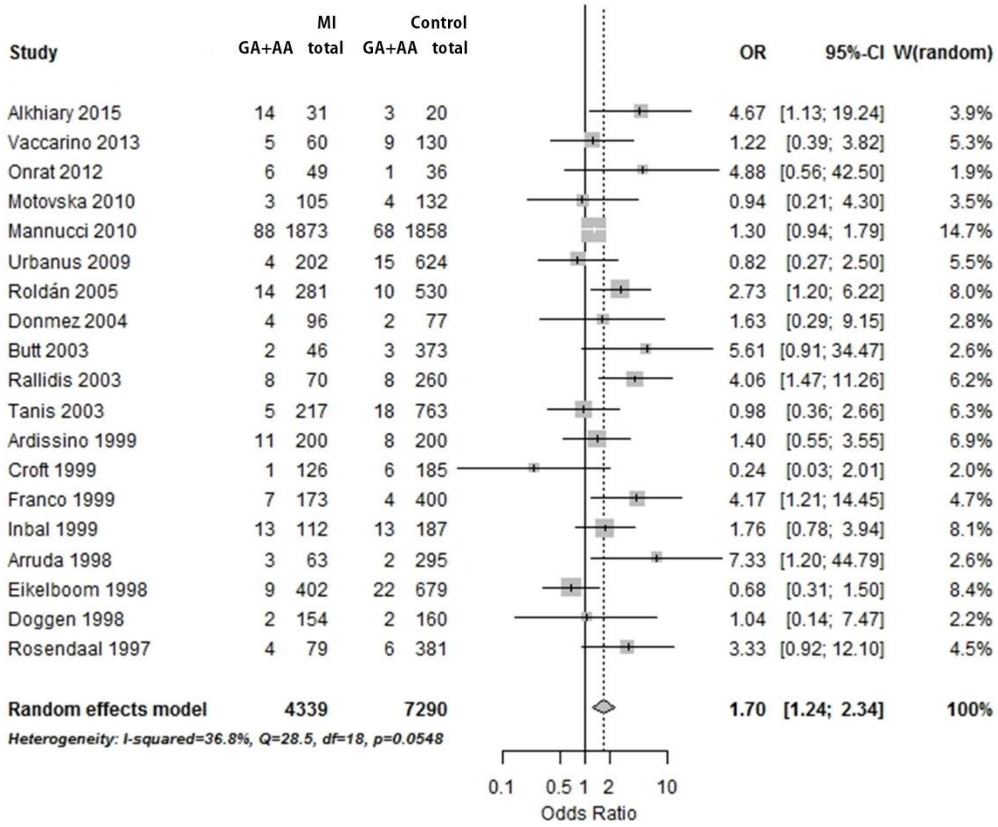

Supplementary Figure S6. Forest plot for subgroup younger than 55 years analysis of association of prothrombin G20210A polymorphism and myocardial infarction risk in a dominant model (GA+AA vs. GG). CI: confidence interval, OR: odds ratio, MI: myocardial infarction.

Recessive model（AA vs. GA+GG, <55）

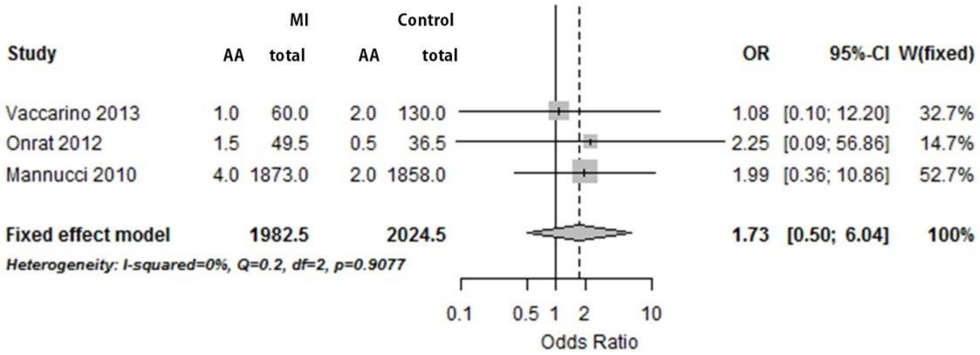

Supplementary Figure S7. Forest plot for subgroup younger than 55 years analysis of association of prothrombin G20210A polymorphism and myocardial infarction risk in a recessive model (AA vs. GA+GG) . CI:confidence interval, OR:odds ratio, MI: myocardial infarction.

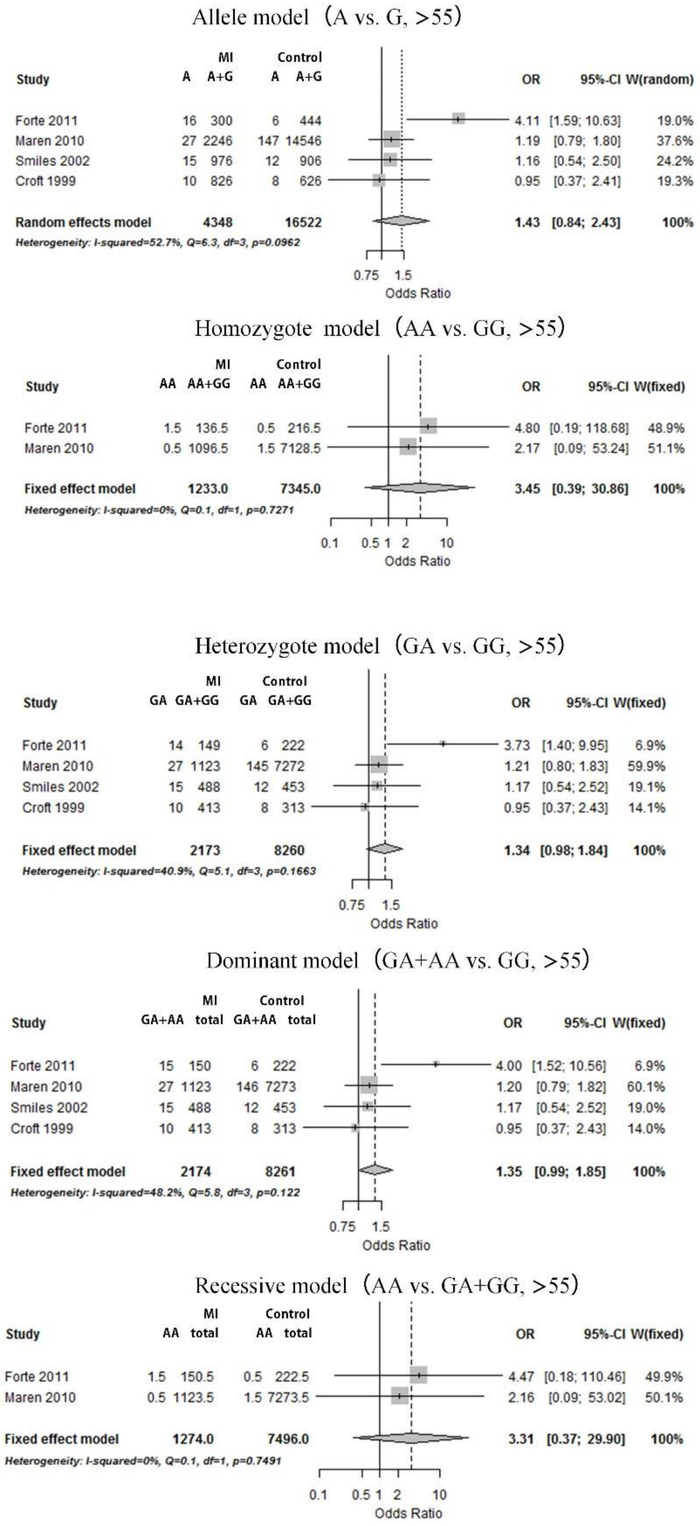

Supplementary Figure S8. Forest plot for subgroup beyond 55 years analysis of association of prothrombin G20210A polymorphism and myocardial infarction risk in allele model(A allele vs. G allele), homozygote model(AA vs. GG), heterozygote model (GA vs. GG), dominant model (GA+AA vs. GG), recessive model (AA vs. GA+GG) . CI: confidence interval. OR: odds ratio. MI: myocardial infarction.

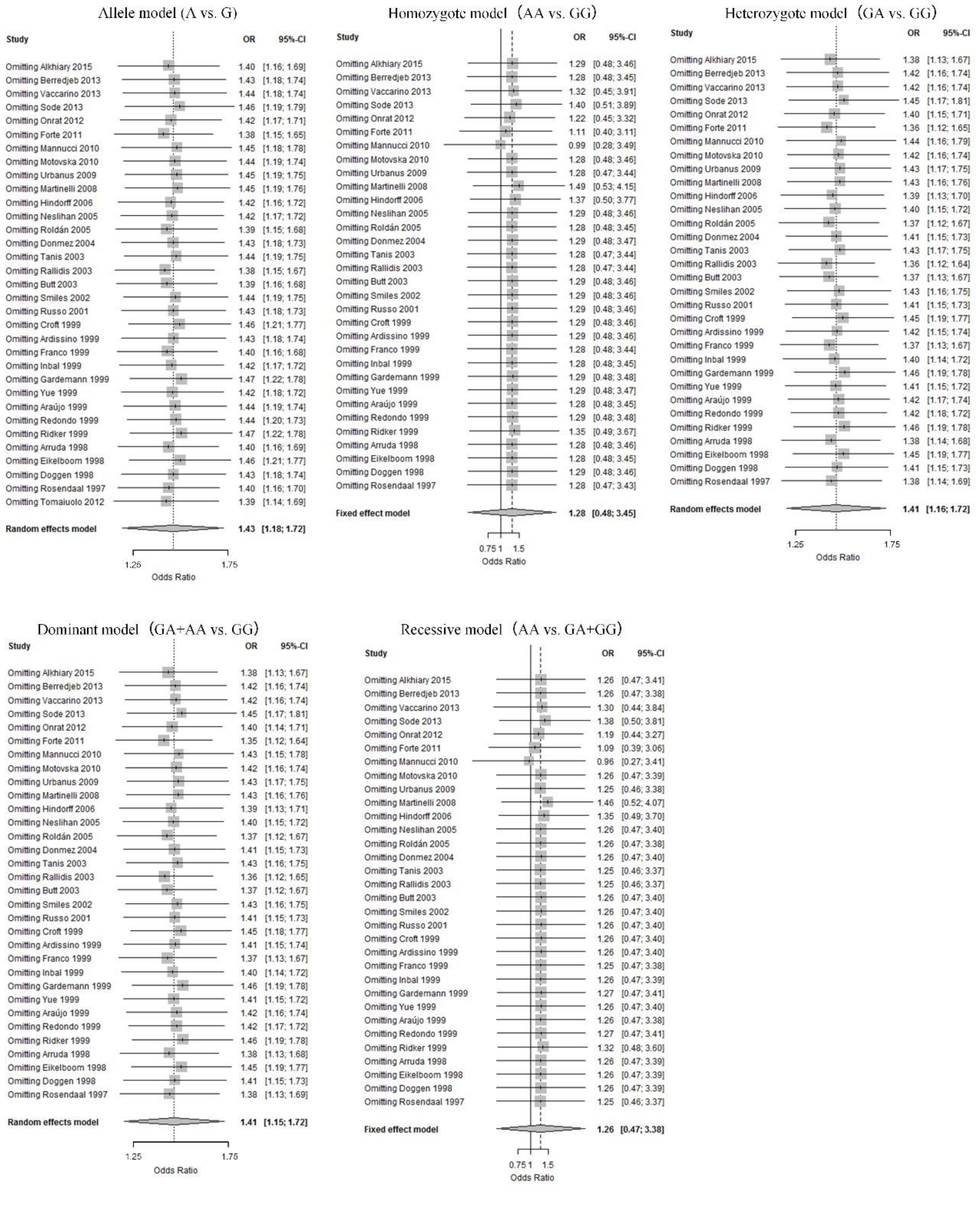

Supplementary Figure S9. Sensitivity analysis to assess the stability of the meta-analysis in all the genetic models (allele model: A vs. G, homozygote model: AA vs. GG, heterozygote model: AG vs. GG, dominant model: AA + AG vs. GG, recessive model: AA vs. GG + AG).

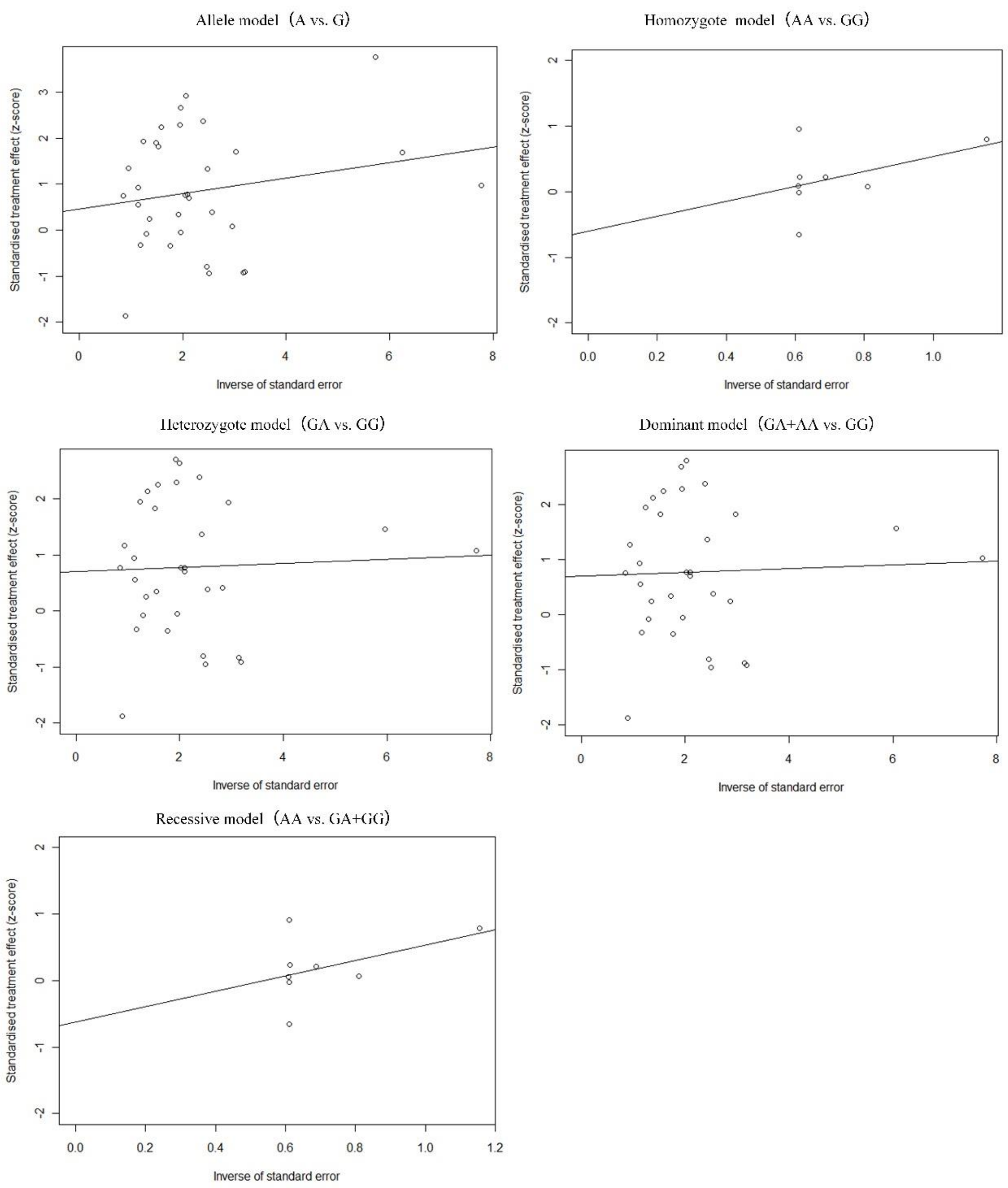

Supplementary Figure S10. Assessment of publication bias on the relationships between G20210A polymorphism and susceptibility to MI with Egger's test in five genetic models (allele model: A vs. G, homozygote model: AA vs. GG, heterozygote model: AG vs. GG, dominant model: AA + AG vs. GG, recessive model: AA vs. GG + AG).

Author	Publication Year	Study Country	Ethnicity	Age of MI patients	Source of Controls	Genotyping method	Genotype frequency GG/GA/AA(number of samples)	MAF
Alkhiary	2015	Egypt	non-Caucasian	＜40 yo	PB	AS-PCR	Case: 17/14/0<br>Control: 17/3/0	Case: 0.23<br>Control: 0.08
Berredjeb	2013	Tunisia	non-Caucasian	＜60 yo	PB	PCR	Case: 97/3/0<br>Control: 195/5/0	Case: 0.02<br>Control: 0.01
Vaccarino	2013	Italy	Caucasian	23–46 yo	matched on age, sex	PCR-RFLP	Case: 55/4/1<br>Control: 121/7/2	Case: 0.05<br>Control: 0.04
Sode	2013	Denmark	Caucasian	20–100 yo	PB	TaqMan assays	Case: 2644/64/0<br>Control: 61966/1305/8	Case: 0.01<br>Control: 0.01
Onrat	2012	Turkey	non-Caucasian	≤45 yo	PB	AS-PCR	Case: 43/5/1<br>Control: 35/1/0	Case: 0.06<br>Control: 0.01
Tomaiuolo	2012	Italy	Caucasian	＜45 yo	PB	real-time PCR	Case: 1795/115<br>Control: 1675/43*	Case: 0.05<br>Control: 0.03
Forte	2011	Italy	Caucasian	55-80 yo	PB	PCR	Case: 135/14/1<br>Control: 216/6/0	Case: 0.05<br>Control: 0.01
Mannucci	2010	Italy	Caucasian	＜45 yo	HB	FRET-PCR	Case: 1785/84/4<br>Control: 1790/66/2	Case: 0.02<br>Control: 0.02
Maren	2010	Denmark	Caucasian	54-68 yo	PB	PCR-RFLP	Case: 1096/27/0<br>Control: 7127/145/1	Case: 0.01<br>Control: 0.01
Motovska	2010	Czech Republic	Caucasian	M≤50 yo，F≤55 yo	HB	PCR-RFLP	Case: 102/3/0<br>Control: 128/4/0	Case: 0.01<br>Control: 0.02
Urbanus	2009	Netherlands	Caucasian	18–49 yo	PB	PCR	Case: 198/4/0<br>Control: 609/15/0	Case: 0.01<br>Control: 0.01
Martinelli	2008	Italy	Caucasian	not restricted	HB	linear-array assays	Case: 289/18/0<br>Control: 298/16/1	Case: 0.03<br>Control: 0.03
Hindorff	2006	USA	Caucasian	＜80 yo	HB	PCR-RFLP	Case: 258/15/0<br>Control: 764/23/1	Case: 0.03<br>Control: 0.02
Neslihan	2005	Turkey	non-Caucasian	not restricted	PB	PCR	Case: 38/4/0<br>Control: 44/2/0	Case: 0.05<br>Control: 0.02
Roldán	2005	Spain	Caucasian	＜45 yo	HB	NR	Case: 267/14/0<br>Control: 520/10/0	Case: 0.02<br>Control: 0.01
Donmez	2004	Turkey	non-Caucasian	＜55 yo	PB	AS-PCR	Case: 92/4/0<br>Control: 75/2/0	Case: 0.02<br>Control: 0.01
Tanis	2003	Netherlands	Caucasian	＜50 yo	PB	PCR-RFLP	Case: 212/5/0<br>Control: 745/18/0	Case: 0.01<br>Control: 0.01
Rallidis	2003	Greece	non-Caucasian	＜36 yo	HB, matched on age, sex	PCR-RFLP	Case: 62/8/0<br>Control: 252/8/0	Case: 0.06<br>Control: 0.02
Butt	2003	Canada	Caucasian	≤50 yo	HB	PCR-RFLP	Case: 44/2/0<br>Control: 370/3/0	Case: 0.02<br>Control: 0.01

Smiles	2002	USA	Caucasian	≥65 yo	PB	PCR-RFLP	Case: 473/15/0 Control: 441/12/0	Case: 0.02 Control: 0.01
Russo	2001	Italy	Caucasian	not restricted	HB	AS-PCR	Case: 233/11/0 Control: 217/7/0	Case: 0.02 Control: 0.02
Croft	1999	UK	Caucasian	＜75 yo	HB	PCR-RFLP	Case: 528/11/0 Control: 484/14/0	Case: 0.01 Control: 0.01
Ardissino	1999	Italy	Caucasian	＜45 yo	HB	AS-PCR	Case: 189/11/0 Control: 192/8/0	Case: 0.03 Control: 0.02
Franco	1999	Netherlands	Caucasian	21-50 yo	PB, matched on race	PCR-RFLP	Case: 166/7/0 Control: 396/4/0	Case: 0.02 Control: 0.01
Inbal	1999	Israel	non-Caucasian	＜52 yo	HB, sex, age matched	PCR-RFLP	Case: 99/13/0 Control: 174/13/0	Case: 0.06 Control: 0.03
Gardemann	1999	Germany	Caucasian	not restricted	HB	PCR-RFLP	Case: 1011/27/0 Control: 478/17/0	Case: 0.01 Control: 0.02
Yue	1999	USA	NR	not restricted	HB, age-matched	PCR-RFLP	Case: 29/3/0 Control: 24/1/0	Case: 0.05 Control: 0.02
Araújo	1999	Portugal	Caucasian	30–82 yo	PB	AS-PCR	Case: 50/2/0 Control: 95/5/0	Case: 0.02 Control: 0.03
Redondo	1999	Switzerland	Caucasian	not restricted	age, sex matched	PCR-RFLP	Case: 176/1/0 Control: 85/4/0	Case: 0.01 Control: 0.02
Ridker	1999	USA	NR	40-84 yo	PB	PCR-RFLP	Case: 392/12/0 Control: 1705/68/1	Case: 0.01 Control: 0.02
Arruda	1998	Brazil	non-Caucasian	＜82 yo	PB	PCR-RFLP	Case: 213/7/0 Control: 293/2/0	Case: 0.02 Control: 0.01
Eikelboom	1998	Australia	non-Caucasian	25-50 yo	PB	PCR-RFLP	Case: 393/9/0 Control: 657/22/0	Case: 0.01 Control: 0.02
Doggen	1998	Netherlands	Caucasian	＜70 yo	HB, age-matched	PCR-RFLP	Case: 540/10/0 Control: 638/8/0	Case: 0.01 Control: 0.01
Rosendaal	1997	USA	NR	18-44 yo	PB, random digit phone	AS-PCR	Case: 75/4/0 Control: 375/6/0	Case: 0.03 Control: 0.01

Supplementary Table S1. Baseline Characteristics of the Studies on the Meta-Analysis. PB: population based. HB: hospital based. PCR: polymerase chain reaction. AS-PCR: allele-specific polymerase chain reaction. PCR-RFLP: polymerase chain reaction-restriction fragment length polymorphism. FRET-PCR: fluorescence resonance energy transfer polymerase chain reaction. NR: no report. MAF: Minor Allele Frequency. * Allele number: G/A

Variables		Overall	Ethnicity		Subgroups	
			Caucasian	Non-Caucasian	≤55 yo	>55 yo
Allele model	FEM OR	1.37	1.36	1.51	1.70	1.36
	(95%CI)	(1.22-1.54)	(1.20-1.54)	(1.06-2.14)	(1.43-2.02)	(0.99-1.85)
	REM OR	1.43	1.40	1.51	1.76	1.43
	(95%CI)	（1.18-1.72)	(1.14-1.72）	(1.00-2.29)	(1.32-2.35）	(0.84-2.43)
Homozygote model	FEM OR	1.42	1.48	ND	1.77	3.45
	(95%CI)	(0.58-3.48)	(0.58-3.78)		(0.51-6.18)	(0.39-30.86)
	REM OR	1.42	1.50	ND	1.75	3.22
	(95%CI)	(0.55-3.65)	(0.56-4.03)		(0.49-6.28)	(0.33-31.07)
Heterozygote model	FEM OR	1.30	1.29	1.51	1.48	1.34
	(95%CI)	(1.14-1.49)	(1.12-1.48)	(1.05-2.15)	(1.20-1.82)	(0.98-1.84)
	REM OR	1.41	1.37	1.56	1.70	1.39
	(95%CI)	(1.16-1.72)	(1.11-1.70)	(1.00-2.41)	(1.24-2.33)	(0.86-2.23)
Dominant model	FEM OR	1.30	1.28	1.52	1.49	1.35
	(95%CI)	(1.14-1.48)	(1.12-1.47)	(1.06-2.16)	(1.21-1.82)	(0.99-1.85)
	REM OR	1.41	1.37	1.57	1.70	1.42
	(95%CI)	(1.15-1.72)	(1.10-1.69)	(1.01-2.45)	(1.24-2.34)	(0.85-2.36)
Recessive model	FEM OR	1.39	1.46	ND	1.73	3.31
	(95%CI)	(0.56-3.42)	(0.57-3.72)		(0.50-6.04)	(0.37-29.90)
	REM OR	1.39	1.48	ND	1.71	3.10
	(95%CI)	(0.54-3.57)	(0.55-3.96)		(0.48-6.14)	(0.32-29.92)

Supplementary Table S2. The odds ratios in both random effect model and fixed effect model. OR: odds ratios; CI: confidence interval; REM: random effect model. FEM: fixed effect model. ND: no data.
